# Physiological and transcriptomic analyses of response of walnuts (*Juglans regia*) to *Pantoea agglomerans* infection

**DOI:** 10.3389/fpls.2023.1294643

**Published:** 2023-12-05

**Authors:** Xiu-Hong An, Ning Wang, Hongxia Wang, Yan Li, Xiao-Yu Si, Shugang Zhao, Yi Tian

**Affiliations:** ^1^ National Engineering Research Center for Agriculture in Northern Mountainous Areas, Agricultural Technology Innovation Center in Mountainous Areas of Hebei Province, Hebei Agricultural University, Baoding, Hebei, China; ^2^ College of Life Sciences, Hebei Agricultural University, Baoding, China

**Keywords:** walnut blight, physiological response, transcriptomic analysis, phenylpropanoid biosynthesis, hormone signal

## Abstract

**Introduction:**

Walnut blight is a serious bacterial disease that affects the yield and quality of walnuts. *Pantoea agglomerans* is one of the main causative agents of walnut blight. However, there have been few studies on the response of walnuts to *P. agglomerans* infection.

**Methods:**

In this study, the soluble sugar, photosynthesis, antioxidant enzyme activities, and secondary metabolites were measured, and the transcriptomic analysis was performed to determine the response of walnut tissue cultures to *P. agglomerans* infection.

**Results:**

After pathogen inoculation, the soluble sugar content decreased, and photosynthesis was inhibited. Antioxidant enzyme (superoxide dismutase and peroxidase) activities and secondary metabolites (phenol and flavonoid) contents increased, especially in the early stages of inoculation. Transcriptomic analysis revealed that the phenylpropanoid biosynthesis pathway is induced after infection, and pathogen infection promotes ABA and ethylene signal transduction and inhibits auxin signaling. In addition, SA and JA-related gene expression was altered after inoculation with *P. agglomerans*, and the FLS- and calcium-mediated disease resistance signaling pathways were activated. Furthermore, our results suggested an involvement of the R-protein RPM-mediated disease resistance pathway in the response of walnuts to bacterial infections.

**Discussion:**

Our findings indicated that phenylpropanoid biosynthesis, hormone signal transduction, and plant-pathogen interaction have key roles in pathogenic inoculation, which provide insights into the molecular mechanisms in the response of walnuts to *P. agglomerans* infection.

## 1 Introduction

Walnuts (*Juglans regia* L.) are one of the four most popular nuts in the world, with high nutritional value and health benefits in their seed kernel (Zhang et al., 2020; Ji et al., 2021). In addition, walnut is an important economic tree, which brings economic benefits and ecological value to the mountainous people and environment in China. Walnut blight is a serious bacterial disease for walnut production worldwide and causes severe damage to the green tissue of the walnut tree, such as bud, leaves, fruits and so on (Mikulic-Petkovsek et al., 2011; Gašić et al., 2022).

*Xanthomonas arboricola* pv. *juglandis* (*Xaj*) was initially identified as the causative agent of walnut blight (Vauterin et al., 1995). It causes brown apical necrosis in walnut fruit and premature fruit drop, leading to significant yield losses (Moragrega et al., 2011). Studies have indicated that *Xaj* may cause primary infection in young walnut fruits, and *Fusarium* spp. and *Alternaria* spp. may participate in the induction of brown apical necrosis, causing secondary infection (Moragrega and Özaktan, 2010). *Pantoea agglomerans* is the causative agent of walnut blight, which causes internal brown apical necrosis in walnut (Yang et al., 2011). *P. agglomerans* belongs to the Enterobacteriaceae family (Cheng et al., 2013; Dutkiewicz et al., 2016) and can infect various hosts. Recently, it has been reported that *P. agglomerans* can cause soft rot disease in cabbage (Guo et al., 2020), leaf shot hole disease in peaches (Zang et al., 2021), necrotic symptoms and dieback in pistachios (Zamorano et al., 2022), blight disease in melons (She et al., 2021), and necrotic disease in mangoes (Gutierrez-Barranquero et al., 2019), jujuba (She et al., 2019), and plums (Li et al., 2020).

The susceptibility of walnuts to bacterial blight depends on the environmental conditions, leafing date, cultivar, soil characteristics, and disease history (Woeste et al., 1992; Olson et al., 1997; Radix et al., 1998; Frutos and Ortega, 2012; Moragrega and Llorente, 2023). The occurrence of walnut blight is more serious in early leafing cultivars and wet years (Mikulic-Petkovsek et al., 2011). Radix et al. (1998) suggested that soil could influence the susceptibility of walnuts to blight by changing the polyphenol content in walnut tissues. Walnut species vary significantly in their resistance to walnut blight, although no species is immune to walnut blight so far (Soltani and Aliabadi, 2010; Frutos and Ortega, 2012). In general, *Juglans sigllata* accessions are more resistant to walnut blight than *J. regia* accessions in the field (Shengke, 1987). Belisario and Zoina (1995) reported that *J. regia* is the walnut species most sensitive to walnut blight. However, there are differences in resistance among walnut genotypes within the same species (Bandi et al., 2015). Jiang et al. (2019b) evaluated the resistance of 18 walnut genotypes, including *J. sigllata*, *J. regia* and their hybrids, against *Xaj* and found that the ‘Qingxiang’ (*J. regia*) variety displayed a relatively strong resistance to blight.

When pathogens infect plants, they produce reactive oxygen species (ROS) (del Río, 2015). To eliminate the damage caused by ROS in plant cells, plants have evolved a complex network of antioxidant systems, including superoxide dismutase (SOD), peroxidase (POD), carotenoids, ascorbate, etc (Foyer et al., 1994; Li et al., 2015). Moreover, phenolics are involved in the defense against pathogens. Differences in the phenolic content of healthy fruits may indicate differences in inherent disease resistance among cultivars. The phenolic content of resistant apple cultivars was higher than that of susceptible cultivars (Petkovsek et al., 2009). However, Mikulic-Petkovsek et al. (2011) showed that the Seifersdorfer cultivar, with a high phenolic content in its fruit, showed extremely high susceptibility to *Xaj*, and the Fernette cultivar, with a low phenolic content, was quite resistant to *Xaj*. Solar et al. (2006) found that seasonal variations in phenolic compounds in annual shoots have a greater influence than phenolic content on the susceptibility of the shoots to walnut blight pathogens. Sagawa et al. (2020) performed proteome analysis using Chandler walnut (*J. regia*) fruits inoculated with *Xaj* and found that the infection enhanced oxidative stress and degradation processes, and biosynthetic metabolism was inhibited in the fruits. In addition, phytohormone-associated defense proteins changed after infection.

Although walnut blight has been reported for many years (Yang et al., 2011; Lamichhane, 2014; Higuera et al., 2015; Chakraborty et al., 2016), previous studies have mostly focused on the isolation of the causative agents and changes in secondary metabolites in infected tissues. The response mechanism of walnuts during host–pathogen interactions is not fully understood. Moreover, most studies have focused on the bacterial blight caused by *Xaj*, and even fewer studies have focused on the response to *P. agglomerans*. However, *P. agglomerans* has been reported as a biocontroller of postharvest fruit diseases to control *Penicillium digitatum* in mandarins, *Penicillium italicum* in oranges, and *Rhizopus stolonifer* in pears and apples (Nunes et al., 2001; Soto et al., 2018). Therefore, it is important to understand the pathogenic potential of *P. agglomerans* to avoid its undesired or dangerous effects on plants and humans when used as a biocontroller. This study aimed at analyzing the physiological responses and transcriptome data from tissue cultures of ‘Qingxiang’ walnuts inoculated with *P. agglomerans* to further understand the plant-pathogen interactions.

## 2 Materials and methods

### 2.1 Plant materials and treatments

Tissue cultures of ‘Qingxiang’ walnuts were used in this study. The walnuts were cultured on Driver and Kuniyuki Walnut (DKW) medium with 1.5 mg/L 6-benzylaminopurine (6-BA) and 0.02 mg/L indole butyric acid (IBA) at 23 ± 2 °C with a 16-h light/8-h dark photoperiod. Tissue cultures were sub-cultured every 25 days. DKW medium contained 1.416 g/L NH_4_NO_3_, 0.265 g/L KH_2_PO_4_, 1.559 g/L K_2_SO_4_, 0.74 g/L MgSO_4_·7H_2_O, 0.149 g/L CaCl_2_·2H_2_O, 1.968 g/L Ca(NO_3_)_2_·4H_2_O, 33.5 mg/L MnSO_4_·H_2_O, 0.39 mg/L Na_2_MoO_4_·2H_2_O, 17 mg/L Zn(NO_3_)_2_·6H_2_O, 4.8 mg/L H_3_BO_3_, 2.5 mg/L CuSO_4_·5H_2_O, 33.8 mg/L FeSO_4_·7H_2_O, 45.4 mg/L Na_2_EDTA·2H_2_O, 0.1 g/L inositol, 2 mg/L glycine, 1 mg/L nicotinic acid, and 2 mg/L VB1.

*P. agglomerans*, a walnut blight pathogen, was isolated from infected walnut tissue. Walnut tissue cultures were inoculated with *P. agglomerans* by stem wound immersion. Samples were collected at 0 (C0), 1 (T1), 8 (T2), and 15 (T3) days post-inoculation (dpi) for physiological data determination and transcriptome analysis. Sixty seedlings were inoculated and three independent replicates were performed.

### 2.2 Measure of soluble sugar content

The soluble sugar content was determined using the Anthrone colorimetric method (Luo and Huang, 2011). The samples were ground, mixed with 80% alcohol, and boiled for 20 min. The supernatant was collected. Next, 80% alcohol was added to the residue, and the mixture was boiled for 20 min. The two supernatants were then combined. The extract was added to Anthrone reaction, and the mixture was boiled for 10 min. After cooling, absorbance was measured at 620 nm in a spectrophotometer.

### 2.3 Determination of Fv/Fm

The maximum quantum yield of PSII (Fv/Fm) was measured using chlorophyll fluorescence imaging systems (FluorCam 7). The seedlings were kept in the dark for 20 minutes before measurement.

### 2.4 Determination of total phenolic content

The total phenolic content was determined using the Folin–Ciocalteu assay (Li et al., 2012). The sample was ground in liquid nitrogen, mixed with 95% ethanol, and centrifuged to obtain the supernatant. The supernatant was added to the Folin–Ciocalteu reaction, allowed to stand for 3 min, and then added to Na_2_CO_3_. The mixture was incubated for 30 min and the absorbance was measured at 760 nm in a spectrophotometer.

### 2.5 Determination of flavonoid contents

The aluminum chloride assay was used to determine the flavonoid content according to Ayele et al. (2022). The samples were ground in liquid nitrogen, mixed with 70% ethanol, and sonicated for 30 min to obtain the crude extract. Crude extracts were added to 5% NaNO_2_ and incubated for 5 min. Then, 10% AlCl_3_ was added, and the mixture was incubated for 1 min. NaOH solution was added, and the mixture was incubated for 10 min. Absorbance was measured at 510 nm in a spectrophotometer.

### 2.6 Transcriptome analysis

Total RNA was extracted from seedlings in the C0, T1, T2, and T3 groups. Transcriptome data were sequenced using the Illumina HiSeq platform (Bolger et al., 2014). Clean reads were assembled and aligned to the walnut reference genome V1.0 (http://xhhuanglab.cn/data/juglans.html) using HISAT2 (Kim et al., 2019). The value of fragments per kilobase of exon per million mapped reads (FPKM) was applied to measure the expression level of genes. Differentially expressed genes (DEGs) were identified using FC≥2 and FDR < 0.01 (Love et al., 2014). The functions of the DEGs were annotated using the GO (The Gene Ontology Consortium 2018) and KEGG databases (https://www.genome.jp/kegg) (Kanehisa et al., 2007).

### 2.7 Validation of RNA-seq by quantitative reverse-transcription PCR

Ten DEGs in the transcriptome were selected, and their expression levels were assessed by qRT-PCR using SYBR Premix Ex Taq (Takara), following the manufacturer’s instructions. The reaction system consisted of 10 μL of SYBR, 1 μL of forward primer (10 μmol/L), 1 μL of reverse primer (10 μmol/L), 1 μL of cDNA, and 7 μL of ddH_2_O. *JrACT2* (XM_018972062.1) gene was used as the loading control. Relative expression level was calculated using 2^-ΔΔCT^ method. All PCR reactions were performed in triplicate. The primers used in this study are listed in **Table S1**.

## 3 Results

### 3.1 Phenotypic analysis of walnut tissue cultures under pathogen treatment

**Figure 1:**
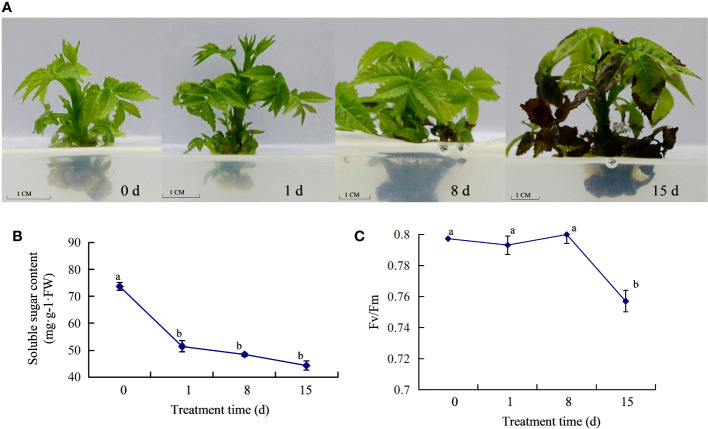
Response of walnut tissue cultures to walnut blight pathogen. **(A)**, Phenotypes of the walnuts inoculated with pathogen; **(B)**, Determination of soluble sugar content; **(C)**, Determination of Fv/Fm.

To test the effect of the walnut blight pathogen on ‘Qingxiang’ walnuts, we analyzed the phenotypic traits of walnut tissue cultures treated with *P. agglomerans*. Compared with 0 dpi, there were no obvious symptoms in tissue cultures at 1 dpi. At 8 dpi, black necrotic spots appeared on the lower leaves of walnut tissue cultures. At 15 dpi, walnut tissue cultures showed more serious symptoms, with complete necrosis of the lower leaves and black stems, and moreover, the upper leaf margin was also necrotic (**Figure 1A**). We determined the soluble sugar content of walnut tissue cultures at different time points. The results showed that the soluble sugar content decreased significantly with inoculation treatment, and the highest content was detected at 0 dpi. The decrease from 0 to 1 dpi was drastic, whereas the decrease from 1 to 15 dpi was moderate (**Figure 1B**). We further determined Fv/Fm in walnut tissue cultures and found no significant difference in Fv/Fm between 0–8 dpi and a significant decreased in Fv/Fm at 15 dpi (**Figure 1C**). These results indicate that walnut blight pathogens affect plant growth and photosynthesis, and plants consume a large amount of soluble sugar in response to the early stages of pathogen infection.

### 3.2 Physiological responses of walnut tissue cultures under pathogen treatment

**Figure 2:**
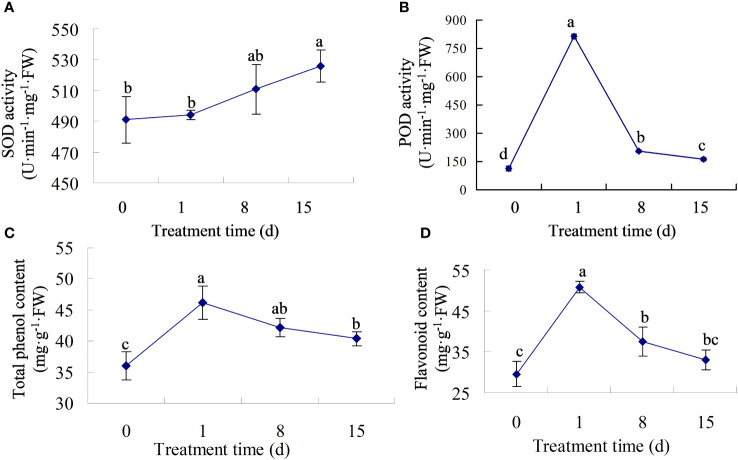
The physiological traits of walnut tissue cultures under walnut blight pathogen treatment. **(A, B)**, Determination of SOD and POD activity. **(C, D)**, Determination of total phenol and flavonoid contents.

We measured the activity of antioxidant enzymes in walnut tissue cultures following pathogen treatment. The results showed that after infection, SOD activity increased gradually, with no significant change in activity at 0–8 dpi, and the highest activity at 15 dpi (**Figure 2A**). However, POD activity first increased and then decreased, reaching its highest value at 1 dpi, which was approximately six times the value at 0 dpi (**Figure 2B**). Previous studies showed that phenols and flavonoids play vital roles in disease resistance. Therefore, we determined the total phenol and flavonoid content in the infected tissues. The results showed that both total phenol and flavonoid contents increased sharply in the early stage of inoculation, reached a maximum at 1 dpi, and then decreased (**Figures 2C, D**), but the contents were still higher than those at 0 dpi, indicating that secondary metabolites may play a major role in the early stage of infection.

### 3.3 RNA-sequencing results

To detect comparative gene expression changes in walnut tissue cultures in response to walnut blight pathogens, transcriptome analysis was performed on seedlings inoculated with *P. agglomerans* at 0 (C0), 1 (T1), 8 (T2), and 15 dpi (T3) using a high-throughput sequencing platform. The quality of the transcriptome data of the 12 samples was assessed. Detailed sequencing and assembly data are presented in **Table S2**. A total of 292,966,049 clean reads were obtained from mapping of the walnut genome sequence. The GC content was between 45.77–46.68%, and the Q30 bases were distributed between 94.01–95.01%. Principal component analysis (PCA) showed that sample C0-3 was clustered with T2 (**Figure S1**), so this sample was removed in the subsequent analysis. There were 2,337 DEGs in C0vsT1 with 962 up-regulated and 1,375 down-regulated genes (**Table S3**). In C0vsT2, there were 1,524 DEGs with 970 upregulated and 554 downregulated genes (**Table S4**). However, in comparison C0vsT3, 7,086 DEGs were found, with 3,742 upregulated and 3,344 downregulated genes (**Table S5**), suggesting that the gene changes were most dramatic in walnut tissue cultures at 15 dpi.

### 3.4 Co-expression patterns of DEGs

**Figure 3:**
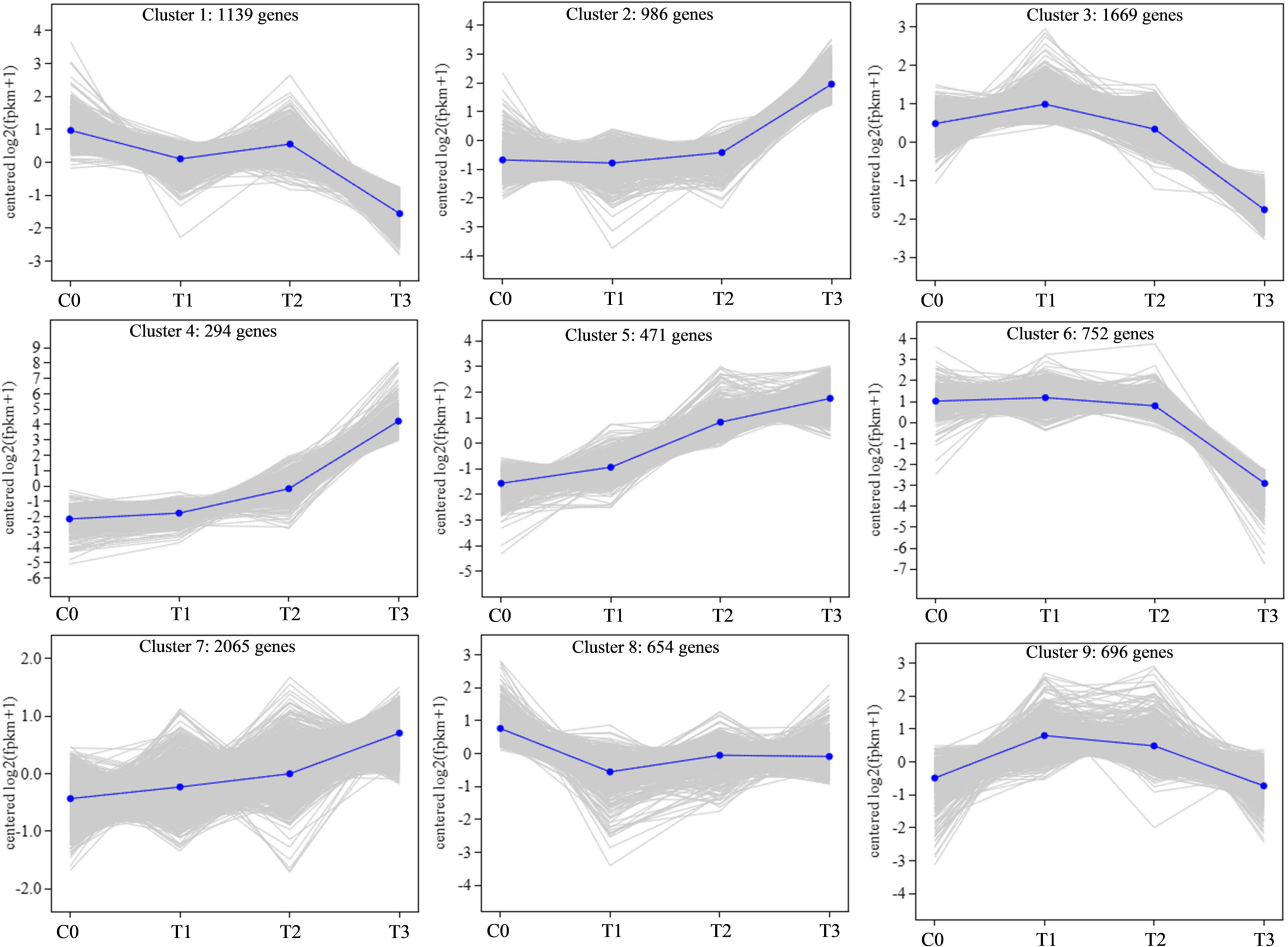
Gene co-expression analysis after pathogen inoculation.

**Figure 4:**
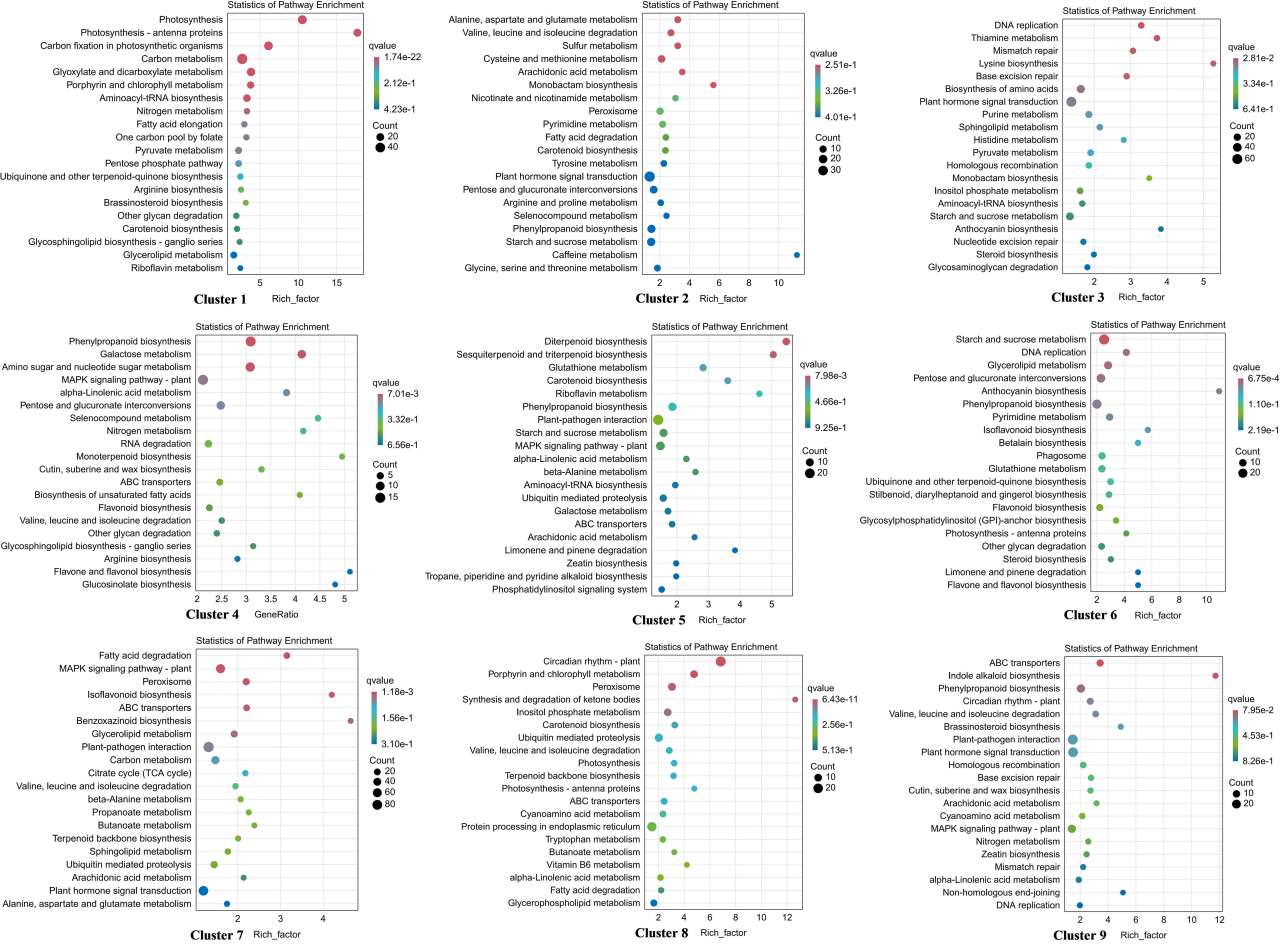
KEGG enrichment of DEGs in 9 profiles. The larger the bubble is, the more the number of DEGs involved. The redder the color of bubbles is, the more significant the enrichment is.

Co-expression patterns were analyzed to detect gene expression trends in walnut tissue cultures during walnut blight pathogen infection. Nine statistically significant profiles were identified (**Figure 3**; **Table S6**). Overall, the expression of genes in clusters 2, 4, 5, and 7 was upregulated after infection, particularly from T2 to T3. Gene expression in clusters 1, 3, and 6 was downregulated after infection, particularly from T2 to T3. Gene expression in cluster 8 first decreased and then increased, and gene expression in cluster 9 first increased and then decreased. In addition, in the early stage of pathogen treatment (C0-T1), the genes in cluster 8 changed significantly and were mainly enriched in the circadian rhythm–plant, porphyrin and chlorophyll metabolism, peroxisome, and inositol phosphate metabolism categories (**Figure 4**; **Table S7**), and the genes were downregulated. Genes in clusters 4 and 5 changed mainly in the middle stage (T1-T2), which was mainly enriched in phenylpropanoid biosynthesis, diterpenoid biosynthesis, sesquiterpenoid and triterpenoid biosynthesis, MAPK signaling pathway-plant, and plant-pathogen interaction categories, and these genes were upregulated. Dramatically upregulated genes in the later stage (T2-T3) were mainly in clusters 2 and 4, which were enriched in phenylpropanoid biosynthesis, MAPK signaling pathway-plant, and amino acid metabolism categories, and the downregulated genes in the later stage were mainly in clusters 1, 3, and 6, which were mainly enriched in photosynthesis, carbon metabolism, biosynthesis of amino acids, phenylpropanoid biosynthesis, plant hormone signal transduction, and other categories. We analyzed the DEGs in clusters 1-6 in which the DEGs were significantly downregulated or upregulated after pathogen inoculation.

### 3.5 DEGs involved in plant hormone signal transduction

**Figure 5:**
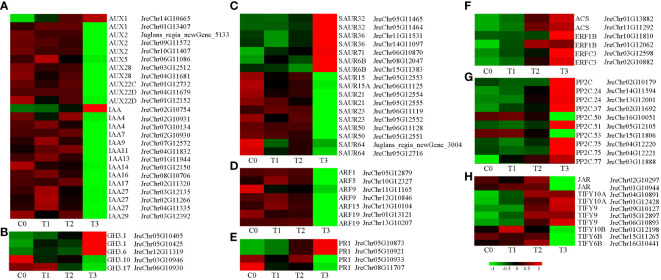
Expression of genes involved in hormone signal transduction after pathogen inoculation. **(A–D)**, Expression of auxin-related genes *AUX/IAA*, *GH3*, *SAUR*, and *ARF*. **(E)**, Expression of SA-related genes. **(F)**, Expression of ethylene-related genes. **(G)**, Expression of ABA-related genes. **(H)**, Expression of JA-related genes. C0, T1, T2, and T3 refer to the 0, 1, 8, and 15 days after pathogen inoculation, respectively. Red and green indicate up- and down-regulation, respectively. Values are shown as log2(fpkm+1), which obtained through gene co-expression analysis.

To analyze the response of hormone signals to *P. agglomerans* infection in walnut tissue cultures, we analyzed the DEGs involved in hormone signal transduction (**Table S8**). First, we analyzed the expression of auxin-related genes. Many auxin response gene expression changed after inoculation, including the early response genes *Aux/IAA*, *SAUR*, *GH3* and the auxin response factor gene *ARF.* Twenty-five *Aux/IAA* were detected, and only two genes, *AUX1* (JreChr14G10665) and *IAA* (JreChr02G10754), were upregulated after inoculation, with the highest expression levels at T3. The expression of the remaining *Aux/IAA* was gradually downregulated or upregulated initially and then downregulated after inoculation, and the expression level was the lowest at T3 (**Figure 5A**). Three *GH* genes, including *GH3.1* (JreChr05G10405 and JreChr05G10425), *GH3.6* (JreChr12G11319) and 7 SAUR genes *SAUR32* (JreChr05G11465 and JreChr05G11464), *SAUR36* (JreChr11G11531 and JreChr14G11097), *SAUR71* (JreChr06G10870), *SAUR6B* (JreChr08G12047 and JreChr15G11383), were upregulated, and all the other *SAUR* and *GH* genes were downregulated (**Figures 5B, C**). In addition, the expression of seven *ARF* genes was downregulated (**Figure 5D**). Ethylene, ABA, SA, and JA are involved in plant stress responses. Therefore, we analyzed the gene expression of these hormone signaling pathways. SA is involved in disease resistance by inducing the expression of *pathogenesis-related* (PR) genes. There were four *PR1* genes; the expression of JreChr05G10873 and JreChr05G10921 was induced, while the other two genes were repressed (**Figure 5E**). 1-aminocyclopropane-1-carboxylate synthase (ACS) catalyses the synthesis of ethylene precursor ACC. Two *ACS* genes (JreChr01G13882 and JreChr11G11292) were upregulated at T2 and T3, and four *ethylene-responsive transcription factor* genes, *ERF1B* and *ERFC3* were upregulated (**Figure 5F**). For ABA signal transduction, ten *protein phosphatase 2C* genes were detected. Two genes, *PP2C.50* (JreChr16G10051) and *PP2C.53* (JreChr15G11806), were highly expressed at C0 and T1, and then decreased following inoculation, whereas the other *PP2C* genes were upregulated, especially at T3 (**Figure 5G**). During JA signal transduction, jasmonic acid-amino synthetase genes, *JAR* (JreChr02G10297 and JreChr01G10944), were highly expressed at C0-T2 and down-regulated at T3. In addition, we obtained nine *TIFY* genes; the expression of *TIFY6B* showed a downward trend, and *TIFY10b* was first upregulated and then decreased. Expression of the other five *TIFY* genes was gradually upregulated after inoculation (**Figure 5H**).

### 3.6 DEGs involved in phenylpropanoid biosynthesis

**Figure 6:**
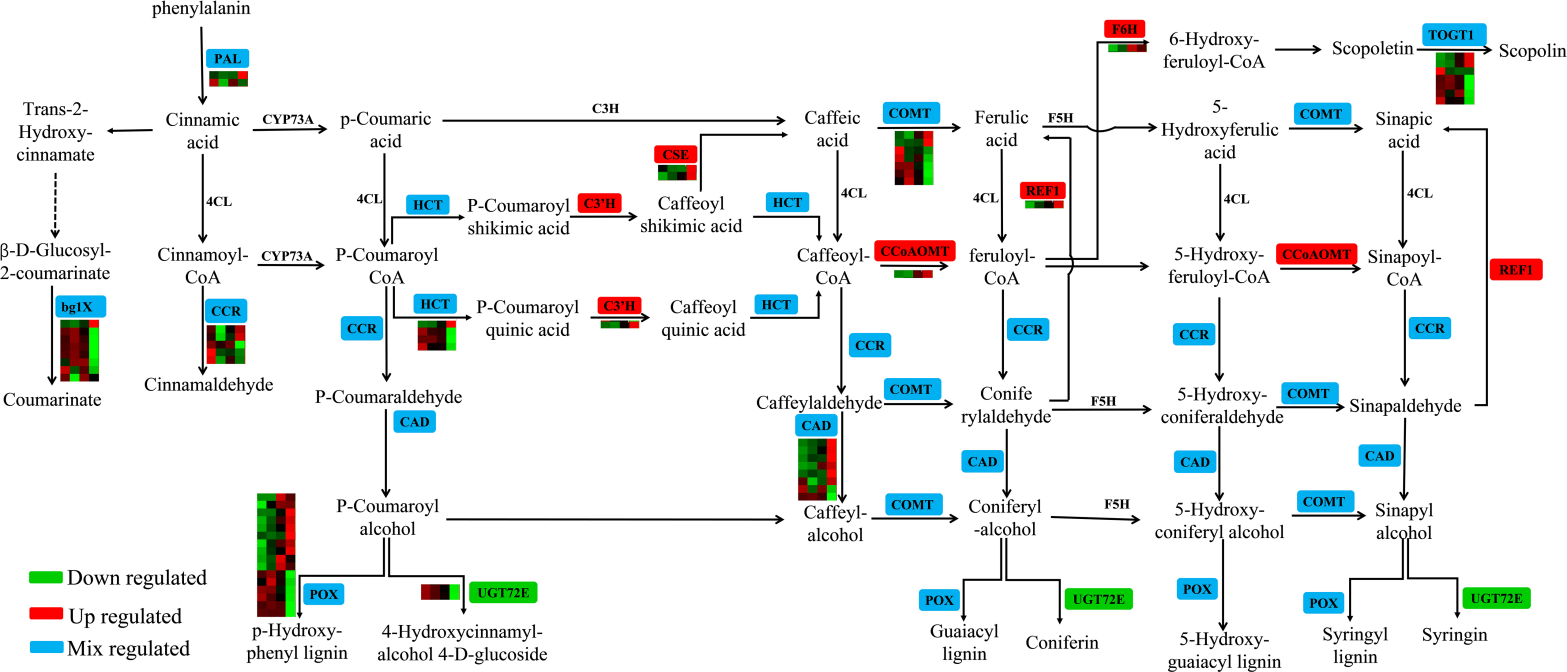
Expression of genes involved in phenylpropanoid biosynthesis. For the heatmap, each row represents a gene, and four columns represent C0, T1, T2, and T3, respectively.

Phenylpropane biosynthesis is an important secondary metabolic pathway in plants that plays an important role in the stress response. There are 65 DEGs involved in the phenylpropanoid biosynthesis pathway in clusters 1–6 (**Figure 6**; **Table S9**). The expression of *CES* (JreChr02G11615 and JreChr04G11602), *C3’H* (JreChr09G12012), *REF1* (JreChr04G11390), *F6H* (JreChr13G10907), and *CCoAOMT* (JreChr05G12881) genes were upregulated by pathogen induction, and the expression levels were highest at T3. *UGT72E* (JreChr01G11984 and JreChr01G11970) was downregulated after infection, with the highest expression at C0. There were 16 *POX* genes; six genes (JreChr03G10690, JreChr04G10636, JreChr03G11462, JreChr15G11746, JreChr14G11423, and JreChr01G13501) had higher expression levels at C0–T2, whereas the remaining 10 genes had low expression at the early stage, and their expression increased gradually after infection. Six *CAD* genes (JreChr09G10469, JreChr10G10465, JreChr09G10465, Juglans_regia_newGene_3795, JreChr06G11577, and JreChr05G10125) were upregulated, with the highest expression at T3, and two *CAD* genes (JreChr08G12114 and JreChr03G10217) were downregulated. However, the expression of most *bglX*, *CCR*, *HCT*, *COMT*, and *TOGT1* genes was higher at C0–T2, and the expression levels were the lowest at T3, indicating that genes involved in the phenylpropanoid pathway were greatly affected by *P. agglomerans*, especially at T3.

### 3.7 DEGs involved in plant-pathogen interaction

**Figure 7:**
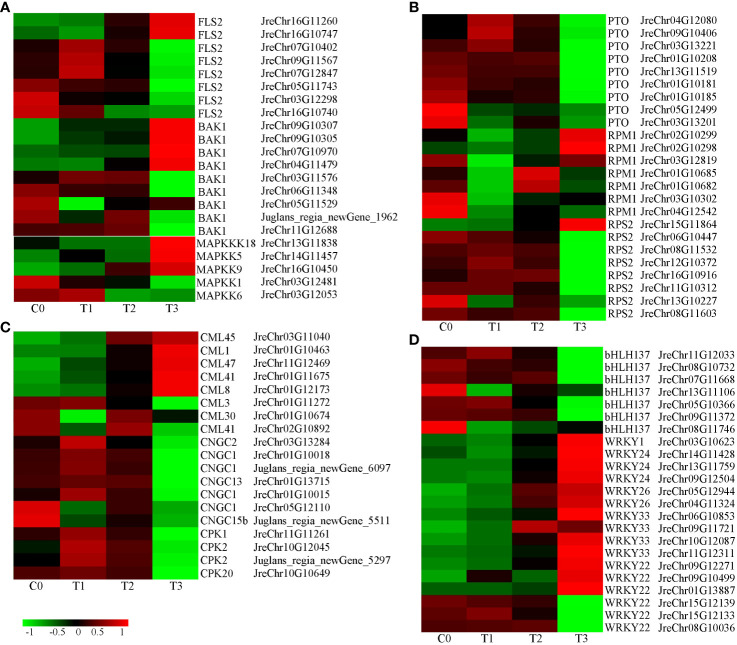
Expression of genes involved in plant-pathogen interaction. **(A)**, Expression of genes involved in flag22-FLS2 mediated disease resistance pathway. **(B)**, Expression of *R* genes. **(C)**, Expression of calcium signaling-related genes. **(D)**, Expression of transcription factor genes *bHLH* and *WRKY*.

Many genes involved in plant-pathogen interactions were differentially expressed (**Table S10**). The receptor-like protein kinase FLS2 recognizes the flagellin protein flg22 to activate pathogen-associated molecular patterns (PAMP)-triggered immunity (PTI). FLS2 interacts with BAK1 to activate the downstream MAPK signaling pathway (Kourelis and van der Hoorn, 2018; Zhou and Zhang, 2020). After infection, eight *FLS2* genes were detected and their expression first increased and then decreased except for JreChr05G11743, JreChr03G12298, and JreChr16G10740. The expression of *BAK1* genes was similar to *FLS2*. *MAPKKK18*, *MAPKK5*, and *MAPKK9* were upregulated, whereas *MAPKK1* and *MAPKK6* were downregulated after inoculation (**Figure 7A**). R proteins recognize different pathogen effectors that activate effector-triggered immunity (ETI). There are three types of *R* genes: *PTO*, *RPM1*, and *RPS2*. Three *PTO* genes (JreChr04G12080, JreChr09G10406, and JreChr03G13221) were upregulated at T1 and then decreased. The other six *PTO* genes were down-regulated after inoculation (**Figure 7B**). Except for JreChr03G10302 and JreChr04G12542, the *RMP1* genes were upregulated in infected tissues (**Figure 7B**). In addition, *P. agglomerans* infection changed the calcium signaling pathway. Cyclic nucleotide-gated channels (CNGC) are Ca^2+^ channels responsible for cytoplasmic Ca^2+^ signaling in plants. *CNGC1* (JreChr05G12110) and *CNGC15b* (Juglans_regia_newGene_5511) were downregulated, the other *CNGC* genes expression first increased and then decreased, with the highest expression observed at T1. *CPK* showed the highest expression at T1. Among *calcium-binding protein* (CML) genes, *CML3* was downregulated, *CML30* and *CML41* (JreChr02G10892) were highly expressed at C0 and T2. *CML1*, *CML8*, *CML41* (JreChr01G11675), *CML45*, and *CML47* were upregulated in infected tissues, with the highest expression at T3 (**Figure 7C**). Furthermore, we analyzed the expression of transcription factors involved in plant-pathogen interactions and found that two types of transcription factor genes were differentially expressed. Seven *bHLH137* genes were downregulated, with the lowest expression observed in T3. Except for three *WRKY22* genes (JreChr15G12139, JreChr15G12133, and JreChr08G10036), the other *WRKY* genes were all upregulated after inoculation, with the highest expression at T3, which showed an opposite trend to *bHLH137*, indicating that these *WRKY* genes may have opposite functions to *bHLH137* in response to pathogen infection (**Figure 7D**).

### 3.8 qRT-PCR validation of DGEs

**Figure 8:**
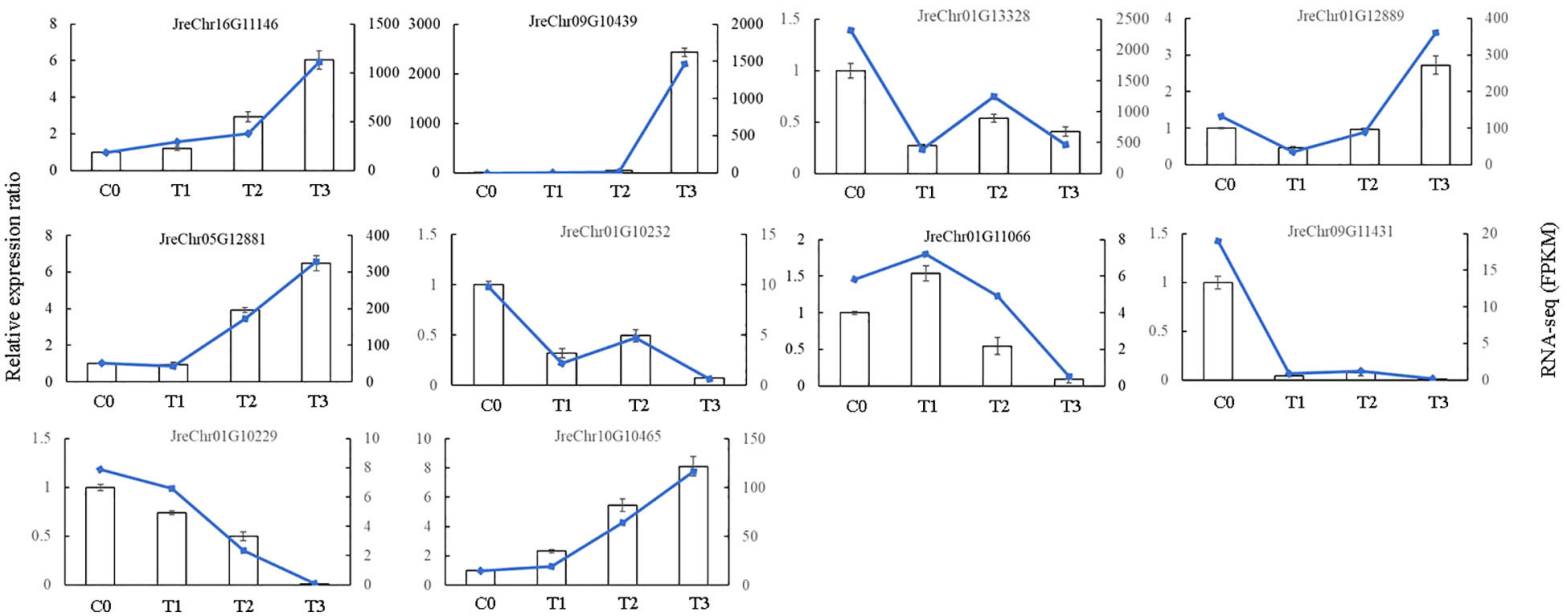
The qRT-PCR validation of selected genes.

To verify the accuracy of our RNA-seq data, we randomly selected ten DEGs in the phenylpropanoid pathway for PCR validation. The results showed that the trend in gene expression obtained by qRT-PCR was consistent with that obtained by RNA-seq (**Figure 8**), indicating that the RNA-seq data were reliable.

## 4 Discussion

Walnut blight is a severe disease in walnuts that can cause serious economic losses. Currently, copper-based biocides are commonly used to control walnut blight. However, these biocides can easily cause bacteria to develop resistance and pollute the environment (Jiang et al., 2019a). Therefore, it is necessary to explore the plant-pathogen interactions in walnuts infected with bacterial blight. In this study, transcriptome data were collected to understand the response mechanisms of walnuts to *P. agglomerans* infection.

When attacked by pathogens, ROS rapidly accumulate in plant cells. To avoid excessive ROS damage to cells, plants have evolved antioxidant systems, including SOD, POD, ascorbate peroxidase (APX), catalase (CAT), etc (De Gara et al., 2003). In maize inoculated with *Fusarium verticillioides* and *Piriformospora indica*, the SOD, CAT, and glutathione S-transferase (GST) activities in the roots significantly increased (Kumar et al., 2009). In Cucumber plants, the activity of antioxidant enzymes such as SOD, POD, and CAT was significantly increased after Cucumber mosaic cucumovirus inoculation (Sofy et al., 2020). Similar results were found in our study; the POD activity increased after *P. agglomerans* infection, especially in the early stage (**Figure 2B**), and moreover, SOD activity increased, although the increase was slower (**Figure 2A**).

More researches proved that the secondary metabolites such as flavonoids and phenols played important roles in plant defense reactions and plant–pathogen interaction (Sofy et al., 2020). Mikulic-Petkovsek et al. (2011) found that the cumulative content of polyphenols in fruits was weakly correlated with walnut blight susceptibility, and the total content of the analyzed phenolic compounds increased after infection with *Xaj*, especially in the walnut cultivars Cisco and Zdole. Petkovšek et al. (2008) found that all the phenolic substances detected, including chlorogenic acid, caffeic acid, p-coumaric acid, rutin, catechin, epicatechin, and quercetin-3-O-rhamnoside, were higher in infected leaves than in healthy leaves. Similar results were found in walnuts, in which the total phenolic content and total analyzed phenolic content of leaves infected with *Xanthomonas campestris* pv. *Juglandis* was higher than that in healthy leaves (Medic et al., 2022). After infection with *V. inaequalis*, the ability to accumulate flavanols differs between resistant and susceptible apple cultivars (Treutter and Feucht, 1990). However, Sierotzki and Gessler (1993) showed that preformed flavan-3-ols and disease resistance were not positively correlated in *Malus* x *domestica* and *Venturia*. In our study, the total phenol and flavonoid content was accumulated rapidly in the early stages of pathogen inoculation (**Figures 2C, D**), although there were no obvious symptoms at this time (**Figure 1**). In addition, transcriptome data analysis showed that the expression levels of many genes involved in the phenylpropane pathway were higher in the early stages of infection (**Figure 6**), suggesting that secondary metabolite pathways may be involved in the response of walnuts to bacterial blight, especially in the early stages. In addition to secondary metabolites and antioxidant enzymes, the soluble sugar content and Fv/Fm changed after pathogen infection and showed a downward trend (**Figures 1B, C**), indicating that *P. agglomerans* infection altered energy metabolism and photosynthesis and then inhibited walnut tissue culture growth.

Plant hormones, such as SA, ethylene, and JA, play important roles in disease resistance and crosstalk. Abdelrahman et al. (2017) found that SA signaling-related genes *PR1* and JA biosynthesis and signaling-related genes *PLDα1* (phospholipase D alpha 1), *OPR* (12-oxophytodienoate reductase), and *JIP23* (jasmonate-induced protein 23 KD) were upregulated in resistant wild *Asparagus kiusianus* stems relative to susceptible *A. officinalis* after *P. asparagi* infection. Sagawa et al. (2020) found that the PR proteins PR1, PR2, PR3, and PR5 significantly accumulated in walnut fruits with *Xaj* infection, and moreover, the ethylene biosynthesis proteins 1-aminocyclopropane-1-carboxylate oxidase (ACC) and S-adenosylmethionine synthase (SAM) were increased. Kong et al. (2020) showed that auxin signaling antagonizes SA signaling by regulating *Pseudomonas syringae* pv. *tomato* DC3000 infection through *Arabidopsis* lateral roots. Zhao and Li (2021) suggested that SA, JA, ethylene, ABA, auxin, GA, cytokinins, and brassinosteroids (BRs) act together to regulate plant-virus interactions. In our study, the expression of most auxin-related genes was higher in the early stage of pathogen infection and decreased in the later stage, while the expression of ethylene- and ABA-related genes showed the opposite trend (**Figure 5**), indicating that *P. agglomerans* infection inhibited auxin signaling and promoted ethylene and ABA signaling, whereas SA and JA signaling may play roles throughout the process of plant-pathogen interaction. Whether these hormones have synergistic or antagonistic effects on the response of walnuts to pathogenic infections requires further investigation.

Plants have evolved a two-tiered immune system that includes the PTI and ETI. Flg22 is a 22-amino acid peptide located at the N-terminus of flagellin, which is recognized by a variety of plants and induces PTI. After *P. agglomerans* inoculation, flg22-FLS2 mediated signaling changed significantly, and more *FLS2* and *BAK1* genes first increased and then decreased (**Figure 7A**), indicating that PT1 was induced in walnut tissues by *P. agglomerans* treatment. Calcium is a secondary messenger that plays an important role in the activation of the plant immune system. Our results showed that the expression of calcium signaling pathway genes first increased or decreased (**Figure 7C**), indicating that pathogen inoculation activated calcium signaling pathways. However, the expression of R-protein genes *PTO* and *RPS* was mainly suppressed at T3, and *RPM* was upregulated at T2 or T3 (**Figure 7B**), indicating that the ETI pathway may be involved in the response of walnuts to pathogen infection. Further studies are needed to determine which R-proteins play roles in the recognition of blight pathogen in walnut.

## 5 Conclusion

Our results indicated that *P. agglomerans* infection affects energy metabolism and photosynthesis, induces the phenylpropanoid biosynthesis pathway, and promotes the accumulation of phenols and flavonoids in walnut tissue cultures. In addition, bacterial infection inhibits auxin signaling and promotes ABA and ethylene signal transduction. FLS- and calcium-mediated disease resistance pathways are activated, and the R-protein gene *RPM* may be involved in the response of walnuts to pathogen infection. These results provide insights into the molecular mechanisms in the response of walnuts to *P. agglomerans* infection.

## Data availability statement

The original contributions presented in the study are included in the article/**Supplementary Files**, further inquiries can be directed to the corresponding author/s.

## Author contributions

X-HA: Formal analysis, Writing – original draft, Writing – review & editing. NW: Formal analysis, Writing – original draft. HW: Funding acquisition, Project administration, Writing – review & editing. YL: Formal analysis, Validation, Writing – review & editing. X-YS: Formal analysis, Validation, Writing – review & editing. SZ: Data curation, Formal analysis, Writing – review & editing. YT: Project administration, Writing – review & editing.

## Supplementary material

Supplementary Figure 1 The PCA analysis of sequencing samples.

Supplementary Table 1 The primers used for qRT-PCR.

Supplementary Table 2 The quality statistic of the sequencing data.

Supplementary Table 3 DEGs in the C0vsT1.

Supplementary Table 4 DEGs in the C0vsT2.

Supplementary Table 5 DEGs in the C0vsT3.

Supplementary Table 6 DEGs in each cluster.

Supplementary Table 7 The KEGG analysis of DEGs in each cluster.

Supplementary Table 8 DEGs involved in hormone signal transduction.

Supplementary Table 9 DEGs involved in phenylpropanoid biosynthesis.

Supplementary Table 10 DEGs involved in plant-pathogen interaction.
