# Supplementary figures and images for: Physiological and transcriptomic analyses of response of walnuts (Juglans regia) to Pantoea agglomerans infection
Source: Front Plant Sci. 2023 Dec 5;14:1294643. doi: 10.3389/fpls.2023.1294643 (PMC10728658; doi:10.3389/fpls.2023.1294643)

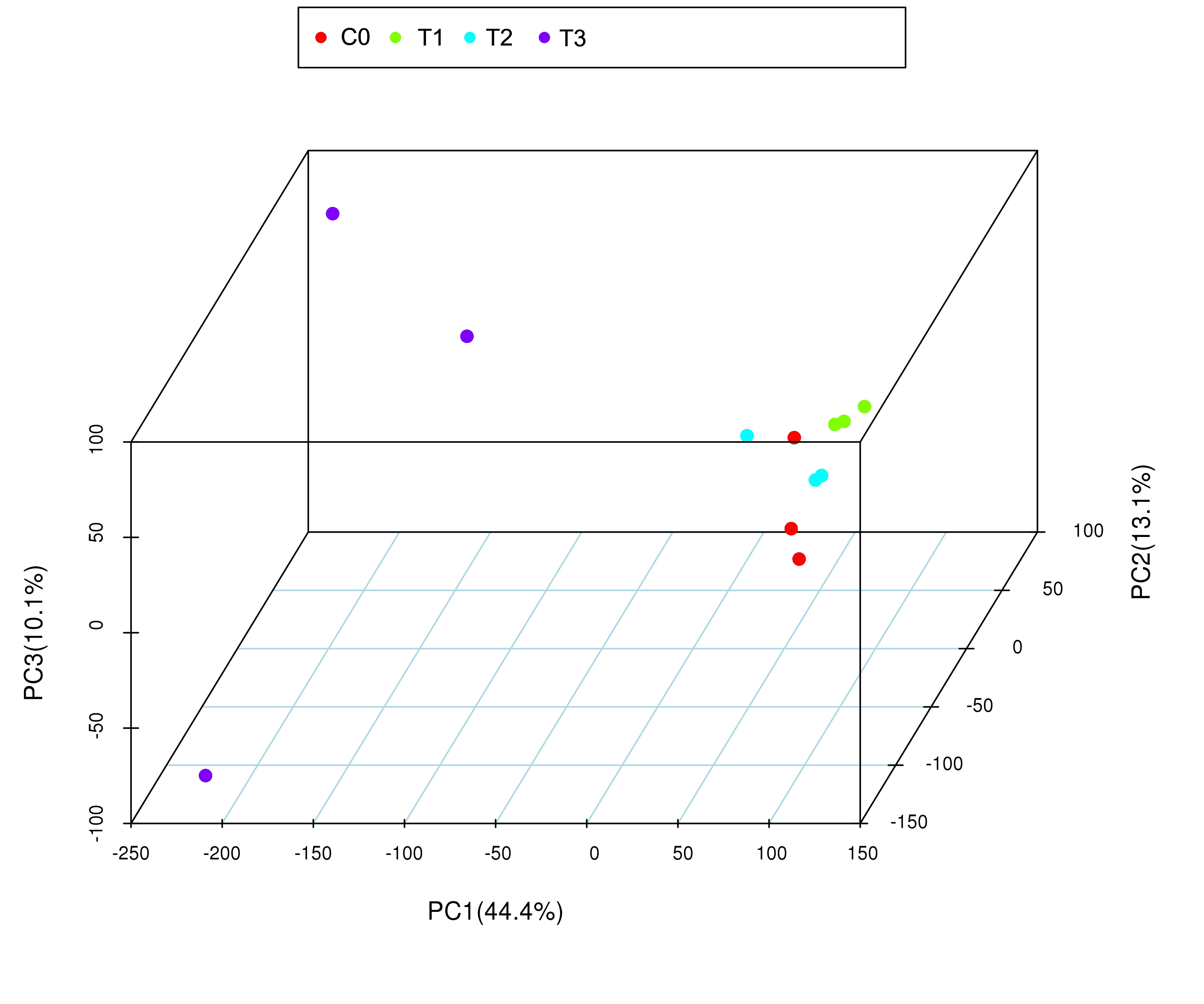

Supplement: Supplementary Figure 1 — The PCA analysis of sequencing samples. [file Image_1.jpeg]
