# Supplementary material for: Physiological and transcriptomic analyses of response of walnuts (Juglans regia) to Pantoea agglomerans infection
Source: Front Plant Sci. 2023 Dec 5;14:1294643. doi: 10.3389/fpls.2023.1294643 (PMC10728658; doi:10.3389/fpls.2023.1294643)
Supplement: Supplementary Table 2 — The quality statistic of the sequencing data. [file Table_2.doc]

| Samples | Clean reads | Clean bases | GC Content | %≥Q30 |
| --- | --- | --- | --- | --- |
| C0-1 | 24,297,525 | 7,257,170,530 | 46.68% | 94.01% |
| C0-2 | 26,267,606 | 7,854,051,032 | 46.39% | 94.82% |
| C0-3 | 22,354,967 | 6,680,810,692 | 46.56% | 94.82% |
| T1-1 | 19,143,059 | 5,723,660,742 | 46.05% | 94.25% |
| T1-2 | 25,017,103 | 7,469,729,432 | 46.22% | 94.84% |
| T1-3 | 23,484,415 | 7,007,676,178 | 46.10% | 94.62% |
| T2-1 | 25,645,654 | 7,658,551,246 | 46.30% | 94.67% |
| T2-2 | 25,830,674 | 7,719,857,794 | 46.01% | 94.58% |
| T2-3 | 28,429,048 | 8,480,697,936 | 46.12% | 94.80% |
| T3-1 | 21,151,608 | 6,326,724,760 | 46.08% | 95.01% |
| T3-2 | 27,825,931 | 8,316,504,090 | 45.77% | 94.83% |
| T3-3 | 23,518,459 | 7,021,942,824 | 46.00% | 94.89% |
